# Effects of pH, lactate, hematocrit and potassium level on the accuracy of continuous glucose monitoring (CGM) in pediatric intensive care unit

**DOI:** 10.1186/s13052-015-0122-x

**Published:** 2015-03-19

**Authors:** Gábor Marics, Levente Koncz, Katalin Eitler, Barbara Vatai, Boglárka Szénási, David Zakariás, Borbála Mikos, Anna Körner, Péter Tóth-Heyn

**Affiliations:** First Department of Pediatrics, Semmelweis University, Budapest, Hungary; Bethesda Children’s Hospital, Budapest, Hungary; Department of Anaesthesiology and Intensive Therapy, Semmelweis University, Budapest, Hungary

**Keywords:** CGM, CGM accuracy, CGM limitations, Tissue perfusion, Intensive care

## Abstract

**Background:**

Continuous glucose monitoring (CGM) originally was developed for diabetic patients and it may be a useful tool for monitoring glucose changes in pediatric intensive care unit (PICU). Its use is, however, limited by the lack of sufficient data on its reliability at insufficient peripheral perfusion. We aimed to correlate the accuracy of CGM with laboratory markers relevant to disturbed tissue perfusion.

**Patients and Methods:**

In 38 pediatric patients (age range, 0–18 years) requiring intensive care we tested the effect of pH, lactate, hematocrit and serum potassium on the difference between CGM and meter glucose measurements. Guardian® (Medtronic®) CGM results were compared to GEM 3000 (Instrumentation laboratory®) and point-of-care measurements. The clinical accuracy of CGM was evaluated by Clarke Error Grid -, Bland-Altman analysis and Pearson’s correlation. We used Friedman test for statistical analysis (statistical significance was established as a p < 0.05).

**Results:**

CGM values exhibited a considerable variability without any correlation with the examined laboratory parameters. Clarke, Bland-Altman analysis and Pearson’s correlation coefficient demonstrated a good clinical accuracy of CGM (zone A and B = 96%; the mean difference between reference and CGM glucose was 1,3 mg/dL, 48 from the 780 calibration pairs overrunning the 2 standard deviation; Pearson’s correlation coefficient: 0.83).

**Conclusions:**

The accuracy of CGM measurements is independent of laboratory parameters relevant to tissue hypoperfusion. CGM may prove a reliable tool for continuous monitoring of glucose changes in PICUs, not much influenced by tissue perfusion, but still not appropriate for being the base for clinical decisions.

## Introduction

Evidence on increased overall mortality in both hypo-, and hyperglycemia has led to an escalating interest in glucose homeostasis disturbances in intensive care units. Pediatric intensive care unit (PICUs) teams are committed to close monitoring of glucose, in view of the vulnerability of the developing central nervous system, and the lack of appropriate clinical signs of hypoglycemia in sedated or unconscious patients [1-7].

Continuous glucose monitoring (CGM) originally developed for diabetic patients [8,9] may be a useful tool for monitoring glucose changes in pediatric or adult intensive care units [10-14]. CGM could be an appropriate additional device of PICU toolbar, but its use is limited by the lack of convincing data on its accuracy at pathological abnormalities causing tissue perfusion disturbance. These clinical settings may potentially lead to temporal shifts between glucose levels of the intravasal and interstitial compartments. Common PICU conditions characterized by diminished peripheral perfusion are hypovolemia, shock, vasoactive therapy with dopamine or noradrenaline, and hypothermia. Studies evaluating the effect of the above situations on the accuracy of CGM measurements has shown controversial results, probable due to the heterogeneity of the critical care population and differences in study design [10,14-16]. Therefore, we tested a set of laboratory parameters that typically change in the PICU either primarily or secondarily in parallel with glucose, measured by CGM and meter glucose measurements to analyze whether any associations could be detected.

The following parameters were tested in our comparative analysis. pH a composite marker of metabolic and/or respiratory insufficiency; serum lactate reflecting on tissue hypoxia; hematocrit (htc), a marker of hemoconcentration and blood viscosity; in addition, serum potassium (SeK) was included as PICU stay can often be affected by potassium abnormalities secondary to renal hypoperfusion and hypofiltration.

The aim of the study was to evaluate the relationship between the accuracy of CGM, characterized by the difference of CGM and meter measurements, and blood pH, htc, lactate and SeK values in the PICU setting.

## Patients and methods

The study was approved by the local research committee and the parents signed informed consent for each child. Pediatric patients (between the age of 0–18 years) admitted to PICU at the 1st Department of Pediatrics of Semmelweis University, Budapest and Bethesda Children’s Hospital, Budapest between 1^st^ February 2012 and 30^th^ June 2013, with an expected stay of at least 3 days were enrolled. Those patients with known diabetes mellitus were excluded from the study. CGM recordings of 40 measurements were analyzed from 38 patients.

Clinical data: mean age (range): 1.3 (0–18) years; gender: 10 females, 28 males; length of PICU stay: mean (range) 21 (1–80 days); 32/38 patients were on mechanical ventilation during CGM measurement; 11/38 needed vasoactive therapy (dopamine, noradrenaline, adrenaline).

### Glucose monitoring system

Interstitial glucose level was monitored by Guardian® REAL Time (Medtronic®, USA) CGM. The flexible platinum Enlite® sensor was inserted in the subcutaneous tissue of the left or right lateral thigh and covered by transparent dressing. Each measurement was started after 2 hours of equilibration period. The initial calibrations were performed at the beginning of the CGM measurement and four hours afterwards. Further calibrations were done regularly at least two times daily. Additional calibrations were repeated as clinically needed. The calibration was not allowed immediately after enteral nutrition or during rapid changes of subcutaneous glucose (more than 1 mg/dL/min, alerted by the display). The range of the glucose measurement by Guardian® Real Time is 40–400 mg/dL. Reference glucose values were obtained from blood gas analyzer (GEM 3000 Premier™, Instrumentation Laboratory®) or point-of-care glucose testing (DCONT Ideal, 77 Elektronika®). Reference blood gas measurements were taken from arterial (77 samples), central venous (184) or capillary (276) sites.

### Data analysis

For this study we used raw CGM data including the calibration’s times and values, that could be exported in comma-separated values format (.CSV) from both Medtronic® CareLink® Professional and Medtronic® CareLink® Personal software. Clarke Error Grid analysis, Bland-Altman analysis and Pearson’s correlation were used for general characterization of the accuracy of CGM. For this, we used both the calibrating values and all the other documented blood gas analyzer glucose results not used for calibration. Clarke Error Grid Analysis was developed to evaluate the accuracy of different glucometers; as its clinical and research application for determination of CGM accuracy is established. It consists of five accuracy zones: A, B, C, D and E. Zone A represents the glucose values that deviate by 20% or less from the reference, or are in the hypoglycemic range (<70 mg/dL) when the reference glucose is also <70 mg/dL. In zone B the deviation is more than 20%, however, without any clinical consequence. Comparison points located in C, D or E require special clinical attention because they could lead to significant harm, if acted upon them The CGM clinical accuracy is the percentage of the comparison points located within zones A and B [17].

The effect of pH, lactate, htc, and SeK levels on the accuracy of CGM were interpreted on line charts (with standard error of mean - SEM - bars) and scatter plots. For the line charts we stratified pH, lactate, htc, and potassium levels in arbitrarily defined ranges. For each range we calculated group’s mean for the difference between reference and CGM glucose levels. On scatter plots each point represented the difference of a glucose pair. The statistical effect was evaluated by repeated measures non-parametric ANOVA (Friedman test, statistical significance was established as a p < 0.05) with STATISTICA 8. Development environment: MATLAB 2010b.

## Results

Altogether 4199 hours of CGM recordings were analyzed from the 40 measurements. Calibration was performed 555 times, 312 of them with archived blood gas records. The mean time between the calibrations was 7.5 hours. Other 225 relevant blood gas records were used for comparison without calibration. In total 537 blood gas records were used for detailed analysis in the study (353 arterial/capillary samples, and 184 venous samples).

**Figure 1:**
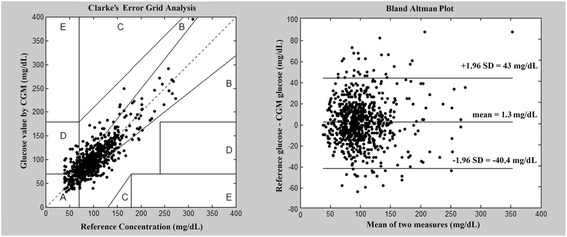
**Left side: Clarke Error Grid analysis.** The clinical accuracy of CGM was 96% as a result of 780 CGM – reference glucose pair analysis. Right side: Bland Altman plot, representing CGM – reference glucose differences in function of mean glucose values.

**Figure 2:**
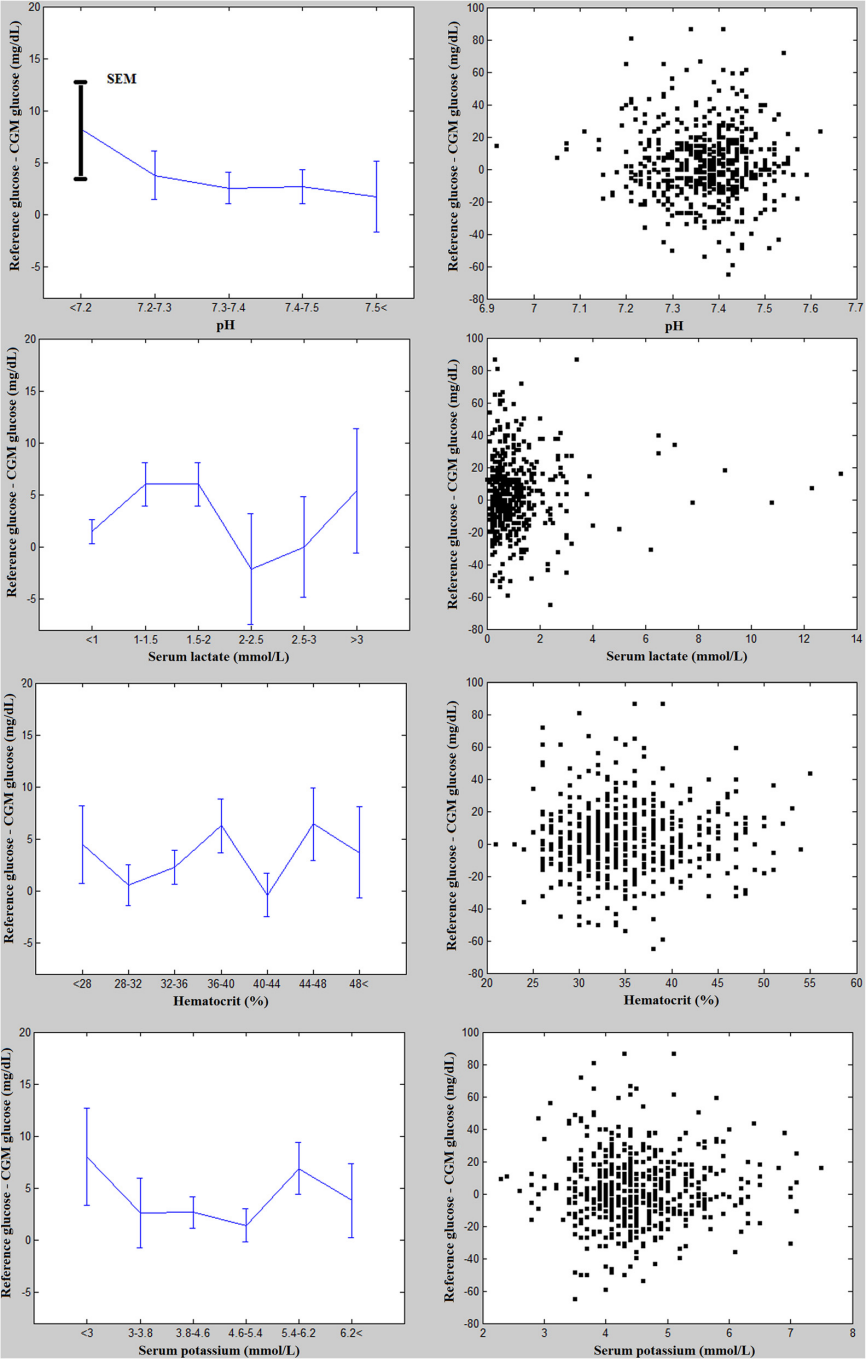
**Left side: Line charts with error bars (standard error of mean [SEM]).** The data points represent the mean difference between the blood gas and CGM glucose concentrations in function of pH, lactate, htc and SeK levels. Right side: Scatter plots. Every rectangle represents the difference between the blood gas and CGM glucose concentrations in function of pH, lactate, htc and SeK levels. In three cases the difference between blood gas and CGM glucose exceeded 80 mg/dL.

The accuracy of the CGM was evaluated based on the calibrating and the relevant blood gas results (n = 780). Pearson’s correlation coefficient was 0.83. By the Clarke Error Grid Analysis 74% of the measurement were in zone A, 22% were in zone B and 4% in zone D. None of the glucose pairs fell in field C or E. The overall clinical accuracy was 96% (zone A and zone B together). The Bland Altman plot showed that the mean difference between reference and CGM glucose was 1,3 mg/dL, 48 calibration pairs exceeding the 2 SD (Figure 1). The average difference of the CGM and the reference (blood gas) glucose measurement was in the range of −2 and 8 mg/dL for all investigated parameters (Figure 2). Although the mean (+SEM) lines seemed to show considerable variability, in the studied range the differences were not statistically significant. Even extreme values of pH, lactate, htc and SeK had no effect on the accuracy of CGM measurement. High levels of error were due to the decreasing number of results in these ranges, but the individual results seen on scatter plots represented a considerably good accuracy of CGM at the extremities (Figure 2). It was, however, notable on scatter plots that CGM had some significant individual error in the normal ranges of investigated parameters. The pH, lactate, htc and SeK did not influence the accuracy of CGM results even if subgroup analysis was performed for arterial, venous and capillary reference measurements.

Table 1 shows the actual pH, lactate, htc, SeK and mean glucose values associated with the ten highest glucose differences compared to the reference values. Data demonstrate that most of these values fell into or near to the normal range. The 2 highest glucose differences were 86 mg/dL with corresponding mean glucose values of 353 and 207 mg/dL. Interestingly, these points were located in the clinically acceptable Zone B of the Clarke Error Grid Analysis (difference from the reference glucose: 23 and 35%).

**Table 1:** **The ten highest glucose differences**

**Blood gas analysis (n = 537)**						
**No.**	**Ref-CGM**	**pH**	**Lactate**	**Htc**	**Se K**	**Mean M**
1	86	7,41	0,3	36	4,3	353
2	86	7,34	3,4	39	5,1	207
3	81	7,21	0,4	30	3,8	132
4	72	7,54	1,3	26	3,6	86
5	67	7,36	0,6	31	4,4	96
6	65	7,2	0,3	34	3,8	135
7	65	7,28	0,5	36	4,5	145
8	−65	7,42	2,4	38	3,5	95
9	61	7,33	0,5	35	4,4	72
10	61	7,41	0,6	28	5,1	96

Ref-CGM = reference-CGM glucose (mg/dL); Htc – Hematoctit (%).

Lactate (mmol/L); SeK – serum potassium (mmol/L).

Mean M - Mean of the two measurements (mg/dL).

## Discussion

Increasing efforts in order to introduce CGM into the intensive care often face the criticism that serum and the subcutaneous tissue are different compartments potentially leading to considerable errors in glucose measurement in cases of disturbed peripheral perfusion [18]. Changes of blood glucose level may appear delayed in the interstitial space when tissue perfusion is compromised. The average delay of about 10 minutes may be significantly prolonged resulting in wide differences between the simultaneously taken blood and subcutaneous glucose values [19]. The need for continuous glucose measurement in the PICU could not allow an increasing reaction time in cases of rapidly changing glucose level.

It is clinically not evident to prove or exclude the problem of inaccurate glucose measurement in peripheral hypoperfusion. Many clinical scenarios could lead to decreased tissue blood flow with other confounding pathogenetic or therapeutic factors, making it very difficult to evaluate the effect on the accuracy of glucose measurements. Simply, there is no homogenous patient group in the PICU characterized by tissue hypoperfusion. We aimed therefore to define a set of simply measurable laboratory parameters which could characterize tissue perfusion, and compare their effects on the accuracy of glucose measurements in the subcutaneous space.

Metabolic acidosis primarily due to anaerobic lactate production is evidently one of the most frequently recognized markers of tissue hypoperfusion. Therefore pH and serum lactate were chosen as key elements of our laboratory tools. In many cases, especially in pediatrics, hypovolemia and secondary hemoconcentration play significant role in disturbed microcirculation. Hematocrit was chosen as an element of our tools based on this approach. Finally, renal function is commonly altered by systemic hypoperfusion, easily detectable from clinical signs such as oligo-anuria, leading to significant morbidity. From practical aspects we have chosen serum potassium as a secondary marker of possibly decreased renal perfusion.

Clearly, all these laboratory parameters could be affected by many other conditions as well. An important consideration for our study was the possibility of measuring these values by a simple bed-side method parallel with blood glucose measurements necessary for CGM calibration. The GEM 3000 blood gas analyzer provides reliable calibrated measurements of all the four above parameters along with blood glucose from venous, capillary or arterial samples, measurements being routinely performed in PICU settings.

With this unique novel approach we could compare 537 pairs of blood and interstitial glucose measurements in an unselected PICU population in function of pH, lactate, htc and Se K. Obviously, a small number of measurements fell in the extreme ranges. The average difference of glucose concentrations was in a considerably narrow range, even at the extremities around 8 mg/dL. pH values < 7.2 and SeK < 3 mmol/L appear to have some effect on the accuracy of CGM measurements, without statistical significance. This could be due to the low number of paired values at these ranges, the scatter plots of all the measurements, however, do not confirm higher dispersion of glucose differences in acidosis, hypokalemia or hyperlactatemia. On the contrary, CGM and reference deviation seems to be relatively low at these states. Three previous studies could not prove the negative effect of vasopressors [16], hypothermia [16] or septic shock [14,15] on the accuracy of CGM, which results are in accordance with our findings.

Some data indicate that there is a lack of agreement between arterial and venous blood glucose measurements in PICU setting [20], suggesting that sampling site could play a significant role in CGM calibration and insulin therapy. To address this issue we performed a selective analysis of our data pairs from arterial, venous and capillary blood samples. We could not demonstrate any difference in CGM accuracy between these groups, but the limited number of cases in arterial and venous groups does not allow us to conclude about the equivalence of the samples.

Scatter plots show a remarkably high variability of glucose differences at the normal range of all four examined parameters reaching 80 mg/dL in three cases. This observation points out to the known limitations of CGM. Based on our results the measurement error showed stochastic distribution rather than exact mathematical nexus, i.e. more measurements - more errors. The question arises which factors are responsible for the inaccuracy of the CGM in PICU settings. A recent study demonstrated that more frequent calibrations (6 hourly) may improve the precision [21]. Other possible, but so far not examined factor is the displacement of the platinum electrode due to the rotation of the PICU patients, being a common anti-decubitus therapy in pediatrics. Furthermore, the Guardian®’s wireless communication might be disturbed by electric interference of the PICU’s electric devices. Very recently, a new generation of CGM monitor was developed (Medtronic Sentrino®), especially for critical care units, with higher accuracy of glucose measurements [22].

Based on the Clarke Error Grid Analysis of our measurements we could state that the clinical accuracy of CGM in our study corresponds to those results in the literature, being in the acceptable range. It remains, however, a right question if conventional CGM is ready for use as a therapeutic guide in the ICU with these limitations of accuracy. It is important to emphasize that CGM is a perfect tool for indicating changes of glucose level in the ICU with a need for confirmation with other methods of blood glucose determination.

## Conclusions

Our results confirm that CGM is a valuable tool in the continuous measurement of glucose levels in the subcutaneous tissue with restrictive inaccuracy for the PICU use. CGM data are very useful for trend analysis in PICU, but the therapy should not be based on CGM values without a confirming reference measurement. The accuracy of CGM is not dependent on the changes of laboratory parameters indicative for tissue hypoperfusion, thus those states typical for PICU patients with disturbed microcirculation do not further limit its suitability.

## Abbreviations

CGM: Continuous glucose monitoring
htc: Hematocrit
PICU: Pediatric intensive care unit
SeK: Serum potassium
SEM: Standard error of mean
